# A Realist Evaluation of Local Networks Designed to Achieve More Integrated Care

**DOI:** 10.5334/ijic.4183

**Published:** 2019-04-05

**Authors:** Lesley Middleton, Harry Rea, Megan Pledger, Jacqueline Cumming

**Affiliations:** 1 Health Services Research Centre, Faculty of Health, Victoria University of Wellington, NZ; 2 Medicine and Integrated Care, South Auckland Clinical Campus, University of Auckland, Otahuhu, Auckland, NZ; 3 Counties Manukau Health, Middlemore Hospital, Otahuhu, Auckland, NZ

**Keywords:** Realist evaluation, local networks, integrated care, New Zealand

## Abstract

**Introduction:**

Not surprisingly given their multi-component nature, initiatives to improve integrated care often evolve to find the best way to bring about change. This paper provides an example of how an evaluation evolved alongside such an initiative designed to better integrate care across primary, community and hospital services in South Auckland, New Zealand.

**Theory and methods:**

Using the explanatory power of a realist evaluative approach, theories of new ways of working that might be prompted by the initiative were explored in: (i) interviews with stakeholders in 2012 and 2015, (ii) online surveys of general practices and local care organisations, and (iii) a purposive sample of ten general practices.

**Results:**

The results highlighted the institutional contexts that led to difficulties in implementing population health initiatives. They also revealed that changes in work practices focussed mostly on activities that improved the coordination of care for individuals at risk of hospital admissions.

**Discussion:**

Multi-component complex interventions can vary in their delivery and be vulnerable to one or more components not being implemented as originally intended. In the case of this intervention, the move towards strengthening local relationships arose when contractual arrangements stalled. Realist evaluative approaches offer a logic that helps unpick the complexity of the relationships and politics in play, and uncover the assumptions made by those developing, implementing and assessing health service changes.

**Conclusion:**

Given the multi-component and evolving nature of initiatives seeking to better integrate care, the realist evaluative emphasis on surfacing early the theories to explain how change is expected to occur helps overcome the challenge of evaluating “a moving target”.

## Introduction

### Problem statement

In recognition that the projected demand for the local hospital was going to outstrip supply, a District Health Board in South Auckland, New Zealand introduced a major change programme focused on shifting the balance of care away from the hospital towards the community [1]. Rather than a single intervention, the programme of change bundled together new financial incentives, new local decision-making networks and a new model of care to better manage long-term conditions. As different activities rose and fell in importance, along with the pattern of intermediate outcomes expected, the external evaluation needed an approach sensitive to the messier realities of health system change. Following the principles of realist evaluation [2], evaluative attention was paid to surfacing the theories expected to drive changes, testing these theories and then offering insights into what was working (or not working) for whom and in what contexts. Initial fieldwork revealed the contexts that meant local financial incentives made little progress while a new model of care made faster progress. Further fieldwork concentrated on exploring the impact of different local areas and practices in taking up a new model of care.

### Background

In 2012, in the context of a long standing interest in delivering care better designed around the needs of the local community, Counties Manukau District Health Board in South Auckland, New Zealand grouped a number of interventions together into a major change programme in order to deliver more care outside of the local hospital. An earlier initiative involving case management of patients who were high users of the hospital was extended to primary care to increase the management of patients in the community [3]. Under the title of the ‘At Risk Individuals’ model of care, payments were provided to general practices to increase patient contact time and connect more with other services via a new information management system. At the same time four geographical localities were created and given responsibility for local planning, design and delivery [4]. These new local networks (which became known as the Localities initiative) were expected to make the delivery of the At Risk Individuals model of care easier and motivate practice improvements by giving greater control over local budgets. Collectively it was hoped that a reduction in hospital bed days would be achieved allowing savings to then be invested in primary care.

Internationally, efforts to better integrate health care have taken many forms [5] often fueled by the belief that if care is better integrated for patients then avoidable hospital admissions can be reduced [6]. The experience of those implementing integration programmes is that the concept itself takes a significant amount of time to clarify in context [7] and ambitious targets of reducing hospital admissions may not always be immediately realised, particularly with respect to the cost savings that can be achieved [8,9]. However, there is good evidence that some initiatives do work to improve patient experience and the quality of care, particularly for those with long-term conditions [10,11,12].

The changes being put in place to increase the role of primary care by this District Health Board were informed by evidence that a greater focus on primary care is associated with better health, a more equitable distribution of health in populations, and lower health costs [13,14,15]. Further, that in order to manage the increasing numbers of the population with long-term conditions, strengthening population screening, monitoring and follow-up in primary care is valuable [16]. Accordingly, the changes within the Localities initiative were informed both by new chronic care models of care [see for example, 17,18], and tools such as primary care-led commissioning [16].

An independent evaluation was commissioned to track progress and evaluate the outcomes achieved from a Strategic Locality Partnership Agreement signed between Counties Manukau District Health Board and the five providers of primary health care within the district. In New Zealand, Primary Health Organisations represent the interests of groups of general practices that generally operate as independent businesses receiving a proportion of their funding from government capitation, and the remainder from patient co-payments. In this district, five Primary Health Organisations representing particular enrolled populations were in operation. In many cases these enrolled groupings cut across the geographical boundaries set up by the Localities initiative.

The Partnership between the five Primary Health Organisations and Counties Manukau District Health Board established a global budget for each locality centred on the expectation that if each locality was able to reduce demand across an agreed set of shared services then the savings would be directed towards innovative primary care services. The partnership agreement was the founding document of the Localities initiative.

Reviews of new contracting arrangements designed to create savings and reduce utilisation have found mixed outcomes. Potential savings can be limited by the time taken to design and implement new contracts, pricing services and agreeing how performance will be assessed [19]. Nevertheless, learning from other experiences seeking to set up global budgets has found such budgets can slow underlying growth in medical spending while improving quality of care [20]. Reviews have found the effectiveness of financial incentives on changing health professional behaviours are likely to be context dependent, particularly as contractual approaches may vary [21]. For these reasons, the evaluation team adopted a realist evaluative approach in order to explore the different contexts and contractual arrangements likely to influence new behaviours within each locality. The starting point of the realist evaluative approach is that the combination of resources offered by a programme (in this instance the Localities initiative) is directed towards altering people’s reasoning. Consequently, the evaluator’s task is to make explicit the theory of how this occurs and then to successively test that theory in order to conclude with an understanding of not only how a programme works, but the conditions that influence its success [22].

## Theory and Methods

The evaluation team undertook an initial assessment of the outcomes expected to be attributed back to the Localities initiative and were struck by the variety of routes being put in place to achieve an outcome concentrated on reducing demand for secondary health care. These routes included new financial incentives via a risk/gain share contract,^1^ new administrative arrangements involving locality leadership teams, a new At Risk Individuals model of care to manage long-term conditions, and new collaborative activities tailored to the particular interests in each locality. Collectively these were expected to achieve a 20% reduction in the standardised rate of use of acute medical inpatient bed days and emergency department services over the next five years. Other outcomes included meeting or exceeding targets such as CVD risk assessments, immunisations and smoking cessation advice, as well as aims to establish primary care as the central focus and coordination mechanism of local health care.

### Application of the realist evaluation framework: the programme theory

Recognising that the Localities initiative was not a fixed thing involving an unchanging set of interventions amenable to a closed system investigation, the following evaluation questions were agreed with the specially created Localities Evaluation Advisory Group:

1. What were the initial theories of how the Localities initiative would achieve change?
2. How is the Localities initiative being implemented, and what are the implications for the initial theories of change?
3. What projects/events/interventions to improve integration most characterise the Localities initiative in health providers’ minds?

Using claims made in planning documents and governance meetings, as well as insights from relevant literature [12,23,24,25,26], three initial programme theories emerged to explain how the Localities initiative would work. The programme theories are couched as context-mechanism-outcome configurations reflecting realist guidance to pay attention to how and why the programme will lead to outcomes and in what kind of settings [27]:

1. The **networks theory.** When health providers are included in local decision-making networks (context), their knowledge about local issues enables them to improve the design and integration of local services (mechanism), which in turn leads to a reduction in demand for secondary services (outcome).
  The early parallels between the Localities initiative and clinical commissioning groups in the NHS, led us to theorise that the proposed risk/gain share contract would employ a similar mechanism of using general practitioners’ (and other allied health providers’) frontline knowledge about patient experiences to improve service redesign [28].
2. The **planned proactive care theory.** When local health providers are resourced to undertake more local coordination activities (context), their confidence in working in a planned proactive way for patients increases (mechanism), resulting in better outcomes for patients and a reduction in demand for secondary care services (outcome).
  The District Health Board had a long-term concern with those adult patients who were very high intensity users of hospital emergency departments [3] and having established a multi-disciplinary team-based approach within the hospital involving a designated “navigator” and assertive follow-up, positive findings led them to consider a more widespread primary care based roll-out of the model [29]. Using the title of the ‘At Risk Individuals’ model of care, local practices were encouraged to adopt a new type of case management for those at risk of secondary care admission. This new model of care drew from insights on the effectiveness of chronic care management programmes [11] and patient self-management approaches [26,30] as well research indicating that patients were less likely to experience poor primary care coordination if their general practitioner knows their medical history, spends sufficient time with them, involves them and explains things well [31].
3. The **relationships theory.** When relationships are strengthened between primary and secondary care health providers, and with social care providers as a result of localities-linked events and activities (context), then increased awareness of who to contact (mechanism) improves the coordination of care for individual patients (outcome).
  A growing theme in the integrated care literature stresses the importance of the softer issues of relationship building [7] in order to create trust between professionals who may otherwise operate with different understandings of what is involved when integrating care. As part of the Localities initiative, hospital based Senior Medical Officers held clinics in each locality which were described by stakeholders as valuable in overcoming misconceptions in primary care as to how hospital specialists want to engage with primary care.

### Evolution of the programme theories

Following the articulation of these initial theories, twelve interviews were held with key individuals associated with the design and implementation of the Localities initiative to identify in more detail how the four localities would work. All interviews were held over the phone using a semi-structured interview guide and lasted up to 40 minutes. Following realist interview guidance to capture the story of the programme being evaluated [32], ten propositions were distilled describing the different ways the Localities initiative was expected to offer value to health providers (See **Box One**). Guidance on developing realist theories suggests that formal theories drawn solely from the literature can end up being too abstract [33], so this initial analysis helped ground the evaluation in changes that were being perceived on the ground, as opposed to concentrating solely on claims made in the planning documentation and the academic literature.

**Box One: Propositions identifying ways Localities initiative would work**

- Health providers will be able to offer input into discussions on how to redesign services to meet specific patients/local community needs.
- Accountability for how resources will be apportioned within each Locality will now be shared with health providers.
- Knowledge about who to contact in the District Health Board will improve the experience for service users.
- Connections with those delivering community health or social services across a Locality will improve the experience for service users.
- Locality General Managers will be a point of contact able to take local ideas for improvement forward.
- Health providers will gain professional development insights from participating in multi-disciplinary teams.
- Service users will benefit from improved collegial relationships within the Locality.
- Health providers will receive more funding as a result of the risk/gain share contract.
- More effective electronic communications will occur from re-designed IT systems.
- Health providers will benefit from quality improvement opportunities linked to initiatives extending the capability of primary care to manage lower acuity events in the community.

Eighteen months into the operation of the Localities initiative a second set of interviews were held with seventeen stakeholders. These interviews included some of the initial designers of the initiative, along with those now responsible for the implementation in each locality and a selection of those providers expected to be influenced by the initiative. Interviews were held over the phone using a semi-structured interview guide. In this guide interviewees were asked to rank and comment on the ten propositions in **Box One**.

Interviews were transcribed and uploaded into NVivo for management and analysis. The results revealed the *most* value from the Localities initiative was coming from improved collegial relationships, and improved knowledge about who to contact in the District Health Board. By contrast, the *least* value was coming from increased accountability for apportioning resources and the potential to receive more funding from the risk/gain share contract. The findings reflected the slow progress on the risk/gain share contract outlined in the Strategic Locality Partnership Agreement. While the District Health Board had hoped to create four entities that would be governing bodies in their own right, the Primary Health Organisations had stronger incentives to maintain what they described as “their own sovereignty”. The result was that the planned move from nominal budget holding (where results were tracked but no funding changed hands), to real budget holding (where funding could change hands) never happened.

**Figure 1:**
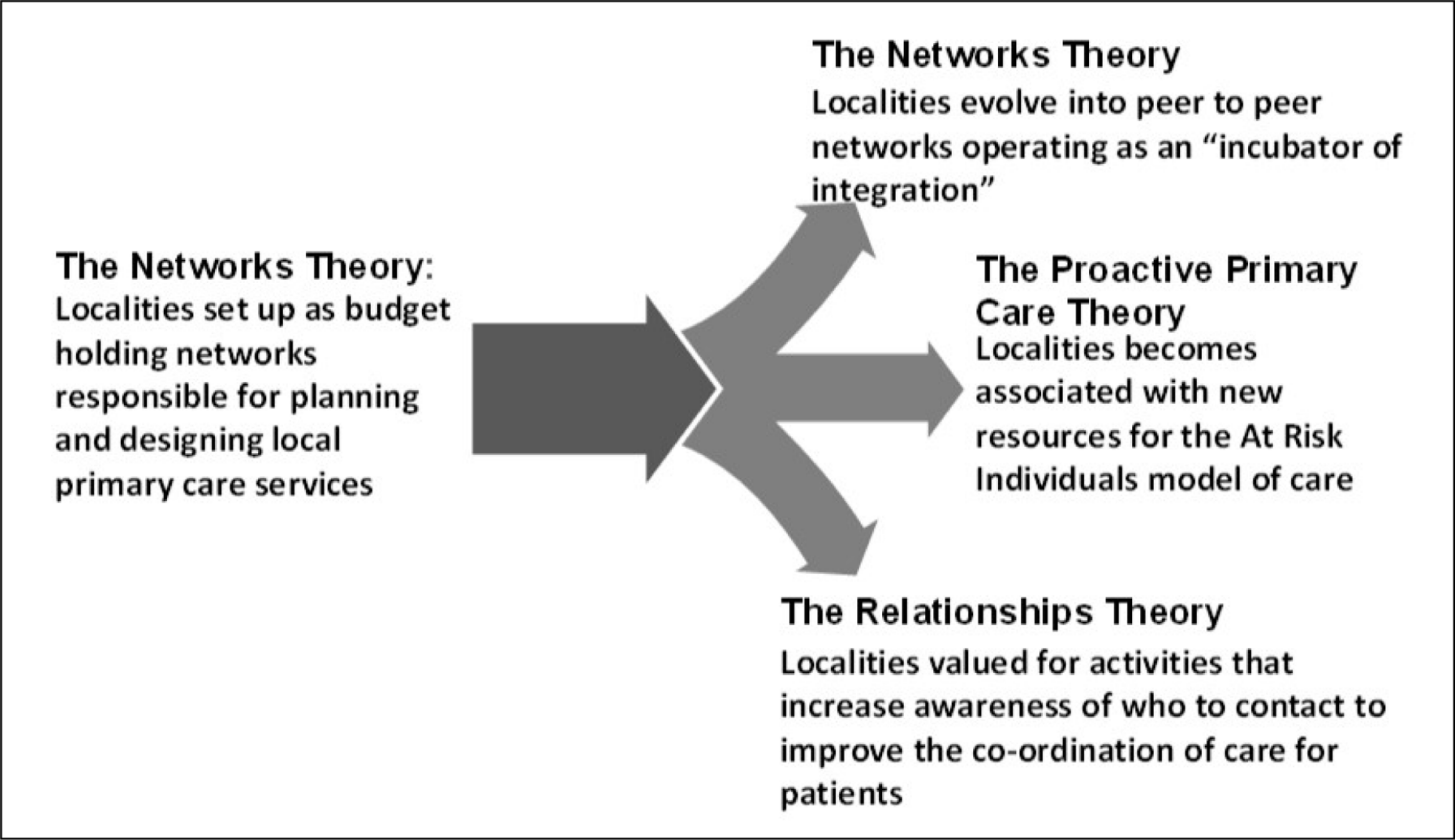
Trajectory of programme theories over the life of the Localities initiative (2012–2016).

At this stage, an interim report was delivered to the Localities Evaluation Advisory Group tracking the evolution of ideas about how the Localities initiative would achieve change. Displayed graphically in Figure 1, the change of emphasis away from new local budget holding networks to a more diverse set of activities led the Advisory Group to agree to a new set of evaluative research questions. These were:

- In what ways and to what extent, are relationships between primary care providers and secondary care providers changing?
- In what ways and to what extent, are relationships between health services and local community care changing?
- In what ways and to what extent, is the At Risk Individuals model of care changing the way general practices work in order to better manage those with long-term conditions?

### Data collection and analysis

In order to answer research questions a and b, two Qualtrics based^2^ on-line surveys were developed, drawing on recent scholarship evaluating integrated care initiatives [34]. These surveys were sent to local general practices (41% response rate) and local care organisations (33.5% response rate). For the survey, local care organisations were defined as those organisations offering care and well-being services locally and were identified by locality managers. These involved, for example, home based services, pharmacies, health advocacy services and mental health services. Questions probed how providers were making sense of the broader integrated care agenda and which relationships were being improved and in what ways.

For the survey sent to local general practices, 55% who replied were general practitioners, and 35% were practice nurses. Responses are reported as percentages as there were large differences in the number of people responding across the survey. Statistical hypotheses were not tested because the sample size was not large especially when broken down across the localities. For comparisons across the different localities, a pragmatic approach was taken in that an absolute difference of 10% was taken as a difference worth mentioning.

To answer research question c (i.e. understanding whether the most significant change project – the At Risk Individuals model of care – was achieving the changes expected), focus groups were held with practice staff in ten general practices. These practices were randomly selected to cover different Primary Health Organisations, different localities and different sized practices (i.e. small practices had less than 200 patients enrolled in the new model of care, medium practices had between 200–399 enrolled and large practices had 400 patients and over enrolled). The grid presented in **Box Two** displays the final sample based on those practices who agreed to participate. The integrated care team alongside PHO D in the grid below covered staff who were not organised in practices but as a wider team supporting walk-in clinics across the PHO. Not all practices in the initial lists from the Primary Health Organisations agreed to participate. In those cases, another practice that met the characteristics in the grid was randomly chosen by the evaluators.

**Box Two: Focus group sample grid**

**Table:** 

	Locality One	Locality Two	Locality Three	Locality Four

PHO A		Small practice Large practice		
PHO B			Large practice	Medium practice
PHO C	Small practice			Large practice
PHO D	Integrated care team (x2)			
PHO E	Small practice		Small practice

The focus groups ranged in size from one through to seven members reflecting that smaller practices often only had one or two members of staff deeply engaged in the At Risk Individuals model of care. A total of 30 general practice staff participated in the groups. The majority of those who participated were practice nurses, though general practitioners, care coordinators and public health specialists were also included in some focus groups.

Three sets of propositions for how the At Risk Individuals model of care delivers value were developed from a review of the At Risk Individuals model of care set-up documents and the literature (particularly the literature on chronic care self-management programmes [26,35,36,37,38,39,40]). These propositions covered:

1. What drives change for patients – practice staff were asked about changes in patient behaviours they observed using six initial propositions of what was expected to happen as a prompt;
2. What drives change for practices – practice staff were asked about the extent to which their practice had changed the way it operated using a further six propositions as a prompt;
3. What drives change for the system – practice staff were asked for their judgements about the feasibility of achieving a number of system wide changes.

Focus group participants were asked about the extent to which these propositions resonated (or not) with their experience. The focus groups were audiotaped and then transcribed to create a qualitative data set that was entered into NVivo and coded against the different patient, practice and system contexts. We refined our understanding of each of our propositions by seeking both confirming and disconfirming evidence from our qualitative dataset. Emerging findings were cross checked within the research team with those who had experience of the day to day delivery of the Localities initiative. Consistent with the realist evaluative approach [2], the study as a whole relied on building a cumulative body of evidence involving different combinations of team members to ensure the trustworthiness of the emerging findings.

The study design was reviewed and approved by the Victoria University of Wellington Pipitea Campus Human Ethics Committee (# 0000021340). All participants had the research explained to them and gave informed consent.

The results presented in the following section provide findings from the surveys which explored how the Localities initiative was working as an ‘enabler’ of improved relationships. The next section focuses on one slice of the findings from the At Risk Individuals focus groups – that is, on what drives change for practices. Further results on the roll out of the At Risk Individuals model of care are presented elsewhere [41]. These further results provide more detail on how being ‘at risk’ was being interpreted by staff. The final section summarises what was happening across the system over the period of the implementation of the Localities initiative.

## Results

### Results from survey of general practices and local care organisations

Initial expectations were that within each of the localities the same project or event may unfold differently because of the different make-up of the populations served, the nature of the organisations within each locality and the different history of established services. Two on-line surveys revealed only small differences between each of the four localities. The lack of variability may be explained by the relatively high numbers of local care organisation respondents who did not align with one locality but were district wide (43%), and the small sample sizes which meant that only an absolute difference of 10% was taken as a difference worth mentioning. Nevertheless, given the considerable early thought that went into grouping providers into geographically distinct communities, the lack of significant differences between each of the localities in the survey results was notable.

In an environment where the central narrative of what the Localities initiative was expected to do shifted from a focus on risk/gain share contracts to local networks acting as “an incubator of integration” [1], providers made sense of the changes by testing whether they improved their knowledge around who to contact to arrange care for individual patients. For general practices, the relationships perceived to have strengthened most in the last three years were those with community pharmacy, home based carer support and mental health services. The relationships most likely to not have changed either positively or negatively (71%) were those with other social services (a category which included housing and welfare support as well as non-government organisations).

Local care organisations, despite already having connections with local general practices, were concerned that awareness of their services and information sharing were not as strong as they could be. Survey results revealed they were looking to Localities-linked activities to support them in their roles as an advocate and coordinator for their clients. Those that chose to provide comments on what influenced their judgements on relationships did refer to specific localities events and working groups, but a few also stressed the relevance of simply being in roles for some time. For example:

“I personally don’t feel my relationships have changed with other providers as I feel I have had good networks in these areas prior to the locality work getting up to speed…. I have witnessed others whose relationships have improved though …including awareness of other providers and networks. Some of this may be caused by the locality work but may also be caused by other factors happening.”

Table 1 presents the results from the general practice surveys when asked about the outcomes that can be achieved from improved integrated care. Table 2 presents the results from the local care organisations. Not surprisingly general practices were most positive about the role of primary care as a coordinating mechanism whereas local care organisations prioritised the importance of local needs being met. Despite the attention paid to reducing the demand for hospital services, there was a high degree of uncertainty from general practice respondents about the potential to achieve this outcome.

**Table 1:** General practice survey results when asked about outcomes expected from integrated care.

To what extent do you agree that integrated care has the potential to achieve the following outcomes?						

n = 29–31/row percents (%)	Strongly agree*	Agree*	Neutral	Disagree	Strongly disagree	Not Relevant

Primary care will become the central focus and coordinating mechanism of healthcare.	32	35	16	10	3	3
Local needs will be better met.	19	55	16	6	3	0
The patient experience of care will be improved.	19	52	16	6	6	0
Inter-professional communication, satisfaction and relationships are improved.	17	53	17	3	10	0
Those in the health workforce will work more at the top of their scope of practice.	17	50	13	10	10	0
Health literacy and patient self-care will improve.	17	31	31	14	7	0
The health status of prioritised groups will improve at a faster rate.	14	41	24	14	7	0
Demand for hospital services will be reduced.	10	39	16	23	13	0
Health disparities for Māori and Pasifika will be reduced.	10	30	37	7	13	3

* The items are sorted on “Strongly agree” then “Agree”.

**Table 2:** Local Care Organisation results when asked about outcomes expected from integrated care.

To what extent do you agree that integrated care has the potential to achieve the following outcomes?						

n = 43–45/row percents (%)	Strongly Agree*	Agree	Neutral	Disagree	Strongly Disagree	Not Relevant

Local needs will be better met.	43	45	9	0	2	0
Primary care will become the central focus and coordinating mechanism of healthcare.	38	42	11	7	2	0
The health status of prioritised groups (for example those with chronic conditions) will improve at a faster rate.	33	35	30	0	2	0
The patient experience of care will be improved.	29	38	31	0	2	0
Inter-professional communication, satisfaction and relationships are improved.	29	53	13	0	2	2
Health literacy and patient self-care will improve.	25	41	30	2	2	0
Those in the health workforce will work more at the top of their scope of practice.	25	45	18	5	5	2
Demand for hospital services will be reduced.	20	41	32	5	2	0
Health disparities for Māori and Pasifika will be reduced.	20	36	34	0	5	5

* The items are sorted on “Strongly agree”.

When participants from general practices provided comments about the concept of integration as whole, perspectives ranged from those who could clearly see the opportunities to achieve better outcomes for patients by being a more active hub of care, to those who had uncertainties over how a better working day could also be achieved within these new expectations. General practice respondents noted workload pressures as the most significant challenge to integrated working (50% strongly agreed and 26% agreed).

Despite workloads in general practice being noted as a significant challenge to integrated care, 97% of the general practices surveyed reported being involved in the At Risk Individuals model of care. Unlike the high percentage of general practices who noted workload pressures as the most significant challenge, local care organisations noted three joint challenges to working more closely together in their local area: (1) systems for sharing data across organisations, (2) differences in how primary, secondary and social care are funded, and (3) awareness of services available (for all three 81% strongly agreed or agreed).

### Results from practice focus groups

This section examines the extent to which a shift towards proactive primary care was occurring based on focus groups in a purposive sample of practices. The At Risk Individuals model of care was implemented and managed by each of the five Primary Health Organisations with the support of the District Health Board, in the expectation that the plans and goals of each locality would be taken into account.

The focus groups revealed that those practices of whatever size that did not hold regular meetings to reflect on what had been learnt from implementing the At Risk Individuals model of care, that tried to absorb the model into their current style of operating and that left nurses to implement it in isolation, were less likely to demonstrate an increased capacity to make connections outside the practice. Those in small practices that tried to absorb the tasks involved in the model into business-as-usual described the struggle involved without having allocated time. For example, those staff who worked in a practice that continued to operate as a walk-in clinic found little support from the rest of the staff “… because it is another extra job in a very busy day”.

Another medium-size practice also reported struggling with incorporating the work into their normal routine, explaining that while they had training to highlight the difference between the At Risk Individuals model of care and an earlier chronic care management programme:

“… when you try and put the training in practice you just couldn’t as there was not time and you just couldn’t allow an hour every week to see a patient for six weeks and spend that time to get the goals and achieve them.” (Medium-size practice participant)

Other practices that had absorbed the At Risk Individuals model of care into business-as-usual were more likely to agree they were resourced for the time involved, often because they had already been doing this work and were now being reimbursed. What characterised these three practices, despite their small size, was the strength of team dynamics they could draw on, built from past experiences delivering care to those with long-term health conditions.

Discussions in one small practice noted that it could be logistically difficult to pass on patients to the one-to-two nurses in the practice whose time was increasingly booked up. Nevertheless, the practice found a way to split responsibilities between the general practitioners who initiated the process and the nurses who followed up, agree specific goals, and develop a care plan. As they explained, “… when it started it was one of the huge issues in our head where were we going to get the time to do all this but it has worked”, [as now]:

“As a doctor I can say OK book 30 minutes with me and I spend that time with the patient and then pass on to the practice nurse … it doesn’t reduce the time I spend having the nurse involved but it increases the quality of what patients receive” (Small-size practice participant).

Larger practices reported adapting to the At Risk Individuals model of care demands by appointing nurse leads, sharing caseloads across nurses and experimenting with different ways of “pulling nurses off the floor” to cover the extra time involved. These staff were also able to draw on a team-based approach to delivering care in partnership with the doctors in the practice. While the At Risk Individuals model of care did involve more work for the nurses, with the right team dynamic they were able to argue for changes to the way the practice allocated time:

“When it first started we were all lost … we did not know what to do … but now as we are going ahead it is more work for us but we are getting more time allocated to do that work as well. We are getting set times. As it is going when we do need more time we are speaking out and we are getting an allocation” (Large-size practice participant).

As being part of a new locality network was expected to influence practices to work in a more planned proactive way for those with long-term conditions, the focus group results revealed different locality contexts had much less influence than the team dynamic within each practice. Those practices prepared to change their organisational processes in order to support nurses to confidently take on new responsibilities for those with long-term conditions, were those most likely to embrace the opportunities to work in a planned proactive way.

### Tracking of secondary care demand

The changes put in place by the Localities initiative were collectively expected to shift the balance of care from the hospital to the community by better integrating services between primary and secondary care, moving from a more reactive to proactive model of care and encouraging the provision of more services in primary care settings. However, an assessment of the high-level trends in secondary care demand across the Auckland region found no evidence of change that could be confidently attributed back to the Localities initiative.

## Discussion

Confirming that integrated care “is not a fixed thing but instead is a fluid state that requires constant amending and adapting” [5], the Localities initiative adapted and evolved and required corresponding shifts in the initiative’s evaluation. Between 2012 and 2015, the Localities initiative shifted from an emphasis on four new local networks charged with reshaping services across a local population to four new networks operating as an “enabler of change” and an “incubator of integration”. Rather than charging new networks with clinical governance decisions, time was spent in local peer-to-peer networks discussing improvements for individual patients and adapting to a new model of care for those with long-term conditions. This shift had implications for how the success of the overall initiative was judged, and the type of new behaviours and engagement expected from different groups of health professionals.

The original Strategic Locality Partnership Agreement was yoked to an ambitious target of a 20% reduction in the projected standardised use of acute medical in-patient bed days and emergency department services over five years. When the Strategic Locality Partnership Agreement was reshaped into a series of enabling activities across the four localities, the policy target remained. However, using the theory-driven realist approach the evaluation sought to assess progress through the intermediate measures of: (i) whether primary care practices were working more proactively for those with long-term conditions; and (ii) the ways in which relationships were improving in order to deliver more integrated care. It is worth reflecting that this smaller scale of experimentation involving a series of enabling activities was forced on the District Health Board by the different power dynamics between the state (in this case the District Health Board) and the non-state (the Primary Health Organisations).

This experience adds weight to broader calls to acknowledge the power dynamics within integrated care [42] and the call for deeper understanding of the facets involved in governing between organisations (collaborative governance) as opposed to within organisations [43]. The fact that the localities’ geographic boundaries rarely matched the boundaries in place around the enrolled population for each Primary Health Organisation, further complicated governance arrangements. Concerns over how the risk/gain share contract would acknowledge the different contributions across five Primary Health Organisations differentially linked to four local communities proved a major stumbling block. In a similar vein, Accountable Care Organisations in the United States are also co-located in geographic areas where other Accountable Care Organisations exist, creating a potential free rider problem if benefit is given to those who improve the health for all people in a given population [44].

From the beginning, the Localities initiative encompassed a bundle of interventions. **Box One** outlined ten original propositions of how the initiative was supposed to work. These propositions included both actions that reshaped services for different local populations and actions that improved the coordination of care for individual patients. As time progressed, the Localities initiative became distinguished by the attention paid to the introduction of a new case management programme (i.e. the At Risk Individuals model of care) and the opportunities to build new working relationships. This finding suggests that of all the propositions in play the ones gaining the most traction were those aiming for improvements for individual patients. Smith’s 2011 analysis of New Zealand’s Primary Health Care directions pointed to unresolved tension between population and patient perspectives within New Zealand primary care. Her assessment was that reform emphasis was placed on the population health gains expected from capitated funding, and less management and policy attention was paid to the development of more integrated primary care services for individual patients [16]. Adaptions of the Localities initiative provide some evidence of an orientation back towards improvements for individual patients.

Multi-component complex interventions can vary in their delivery and be vulnerable to one or more components not being implemented as originally intended [45]. Recent work summing up the emerging achievements of new vanguard models of care in England draws attention to the benefits of building relationships before new ways of working are formalized through organisational or contractual changes [46]. In the Localities case, the move towards the benefits of strengthening local relationships arose when contractual arrangements stalled. Realist evaluative approaches offer a logic that helps unpick the complexity of the relationships and politics in play [47] and uncover the assumptions made by those developing, implementing and assessing health service changes [48]. In the case of the Localities evaluation, building an understanding of the behavioural landscape of professionals [49], offered value, despite the major shifts in *what* was happening to drive change. The key recommendations from the evaluation were to:

- Temper the expectation that the creation of four localities would reduce demand for secondary care and be more targeted about which local relationships need to be improved in order to benefit which groups of patients. The experience confirms the challenges of the field of integrated care, which can easily be saddled with ambitious business cases claiming significant effects when the reality is that behavioural changes are much more nuanced [8].
- Acknowledge the different contexts for local general practices compared to local care organisations. Local care providers were looking to be affirmed as an advocate and coordinator of care for their clients, while general practices wanted to be confident their time was being spent strengthening the local connections most likely to improve individual case management for their patients. These organisational contexts were more influential than the local contexts in each of the four geographical localities.
- Strengthen the implementation of the At Risk Individuals programme by supporting the growth of team-based cultures within primary care practices. Further research is planned on how patients are reasoning differently in order to support decisions on who is enrolled in the programme.

### Strengths and limitations

The strength of this evaluation centres on the application of the realist approach. Recognising that the Localities initiative is a social programme involving human decisions and actions, attention was paid to identifying how change is expected to occur, and then testing the extent to which these theories were borne out. A recent realist evaluation of two integrated care initiatives tried to retrospectively identify the context, mechanism and outcomes in play but struggled to identify the relationships between them [50]. This evaluation has made greater progress in surfacing testable theories of change, but confidence in the findings needs to be tempered by the partial nature of the evidence collected with respect to (i) the survey response rates and (ii) the sheer diversity of indicators where we might expect to see change. The smallness of the sample size for each locality may also have masked some local differences.

## Conclusion

The Localities initiative demonstrated an agility in adapting to the complex web of incentives around primary health care in New Zealand, but the consequence was that attributing what was done to earlier defined metrics of success was difficult. The realist logic of enquiry regards programmes as “theories incarnate” [22]. Surfacing these incarnate theories early helped identify which components of the Localities initiative were rising or falling in importance and was vital to ensuring the evaluation evolved to concentrate on where results could add the most value.

## Footnotes

In risk/gain share contracts, gains and losses are calculated according to the expected costs of delivering care to a defined population with any difference in the actual costs being shared with the contracted health providers. The assumption is that any gain in costs that has occurred comes from efforts by the provider to keep their population healthy. In the Localities initiative, the expectation was that funds that would have been used for extra hospital staff/resources would now be invested in primary and community settings to buy additional services.

Qualtrics is the name of the web-based survey tool used to collect information via sending participants an on-line link.
